# Gain +1 or Avoid −1: Validation of the German Regulatory Focus Questionnaire (RFQ)

**DOI:** 10.1186/s40359-017-0207-y

**Published:** 2017-12-19

**Authors:** Bjarne Schmalbach, Markus Zenger, Roy Spina, Ileana Steffens-Guerra, Sören Kliem, Michalis Michaelides, Andreas Hinz

**Affiliations:** 1 Department of Psychology, University of Münster, Münster, Germany; 2 Faculty of Applied Human Studies, University of Applied Sciences Magdeburg-Stendal, Stendal, Germany; 3 Integrated Research and Treatment Center (IFB) AdiposityDiseases - Behavioral Medicine, Medical Psychology and Medical Sociology, University of Leipzig Medical Center, Leipzig, Germany; 4 Department of Psychology and Counselling, University of Chichester, Chichester, UK; 5 Criminological Research Institute of Lower Saxony, Hannover, Germany; 6 Department of Psychology, University of Cyprus, Nicosia, Cyprus; 7 Department of Medical Psychology and Medical Sociology, University of Leipzig, Leipzig, Germany

**Keywords:** Regulatory focus, Decision making, Motivation, Psychometric properties, Questionnaire

## Abstract

**Background:**

The Regulatory Focus Questionnaire (RFQ) assesses regulatory promotion and prevention focus, which represent orientations towards gains or losses. The main objective of this study was to examine the psychometric properties of the newly translated German version.

**Methods:**

A sample of 1024 participants answered the questionnaire and several related instruments. We used an online survey tool to collect this data. Data analysis was conducted using methods of exploratory and confirmatory factor analysis in SPSS and AMOS.

**Results:**

The RFQ displayed acceptable reliability, while its correlations with other, related psychological constructs indicated good validity. Factor analysis showed good fit for a two-dimensional model. Tests of measurement invariance revealed clear evidence for metric invariance while scalar invariance remained uncertain. Differences in regulatory focus based on sociodemographic characteristics are reported and discussed.

**Conclusions:**

Overall, the RFQ can be recommended for application in fields dealing with motivation and goal attainment in a broad sense.

## Background

Regulatory focus theory (RFT) is a goal pursuit theory that categorizes individuals’ thoughts and behaviors in terms of an orientation towards gains and losses [1–4]. The *promotion system* focuses on the attainment of a desired state whereas the *prevention system* centers on the avoidance of undesirable states. Accordingly, promotion-oriented individuals seek to make gains, seize opportunities, and take risks in order to advance in their pursuits towards ideals. In contrast, prevention-oriented individuals aim to minimize risks, maintain a given status quo, and remain vigilant against potential threats to oughts. These tendencies influence the processing and usage of information and decision making on many levels, and therefore play an important role in several fields of psychological research such as motivation, attitude, persuasion, and leadership, among others [5–10]. Furthermore, considering that specific regulatory focus states can be easily primed, applications of this theory are abundant and diverse [11]. The regulatory focus systems are rooted in specific neural components, as indicated by neural correlates that have been identified, including an activation of the amygdala, the anterior cingulate cortex, and the extrastriate cortex [12]. Additionally, promotion focus relates to an activation of the right prefrontal cortex while prevention focus correlates with an activation of the left prefrontal cortex [13].

Molden, Lee, and Higgins [14] argued that the regulatory focus system is orthogonal to the approach-avoidance system. Proposing a 2 × 2 model, they demonstrated how in approaching a positive end state, individuals can either approach gains (promotion) or approach non-losses (prevention). Similarly, in avoiding a negative end state, individuals can either avoid non-gains (promotion) or avoid losses (prevention). For example, one can strive for (or approaching) a positive end state of health by either exercising regularly to reap the benefits (gains, i.e. promotion) or by not smoking or drinking in order to not lose the health status one already possesses (non-losses, i.e. prevention). On the other hand, one can strive to avoid the negative end state of sickness with the exact same behavior, in a reframed setting: One exercises to avoid missing out on the positive results (non-gains, i.e. promotion) and avoids smoking and drinking in order not to experience the negative effects associated with those behaviors (losses, i.e. prevention). Although the regulatory focus system is theoretically orthogonal to the approach-avoidance system, studies comparing regulatory focus measures with approach-avoidance measures have shown that these two constructs are in fact moderately correlated [15]. As noted by Haws, Dholakia, and Bearden [16], the two regulatory focus measures that are being used most frequently in psychological research are the *Regulatory Focus Questionnaire* (RFQ) by Higgins and colleagues [17] and the *General Regulatory Focus Measure* (GRFM) by Lockwood, Jordan, and Kunda [9]. Summerville and Roese [15] found stronger associations with *Behavioral Inhibition/Approach Scales* (BIS/BAS; Carver & White, 1994) for the GRFM than they did for the RFQ and stronger correlations for promotion than for prevention focus.

There have been a number of attempts at establishing a German measure of regulatory focus: Several translations of the RFQ have been employed by researchers in the past (e.g., [18–21]). However, none of the conducted studies reported detailed psychometric properties – especially the factor structure was never discussed. As this is a very central step in investigating the validity and therefore the theoretical soundness as well as the practical applicability of a scale, it should not be skipped. The GRFM has also been translated and applied before [22], but there is again no discussion of factorial structure. Finally, Fellner, Holler, Kirchler, and Schabmann [23] created a new scale that seeks to address the short-comings of the RFQ and the GRFM, but does only achieve mediocre factorial validity.

### Aims of the study

The present study seeks to validate the newly translated German version of the RFQ. Specifically, it aims to a) investigate psychometric properties including item characteristics and reliability, b) confirm the two-factorial structure proposed by Higgins and colleagues [17], c) examine validity towards related psychological constructs, and d) analyze measurement invariance as well as differences in promotion and prevention focus based on sociodemographic variables.

Considering the moderate correlations found between promotion focus and behavioral approach, and between prevention focus and behavioral inhibition, similar correlations are expected in the present study. Furthermore, regulatory focus (mostly promotion focus) plays an important role in predicting work-related outcomes [24]. Two other constructs that significantly predict work-related outcomes are core self-evaluations and the Big Five, and therefore, the relationships between the RFQ and the subscales of the *Core Self-Evaluation Scale* (CSES; [25]) and the *Big Five Inventory-10* (BFI-10; [26]) were examined. Correlations of regulatory focus and the Big Five have been shown by previous research, such as openness [27, 28]. Individuals with high promotion focus look for opportunities and seek to maximize gains, this implies a necessity for openness to new experiences. In this line of argument, we also expect a moderate association of promotion focus with extraversion. In contrast, individuals with prevention focus, want to maintain vigilance and avoid losses, therefore a positive association with conscientiousness and neuroticism is expected.

As regulatory focus relates to self-regulation high correlations with the CSES, which contains among others self-efficacy and self-esteem, are also expected. Hazlett, Molden, and Sackett (2011) have shown that promotion-oriented individuals tend to be optimistic, whereas prevention-oriented individuals favor pessimism. For this reason, the revised *Life-Orientation-Test* (LOT-R; [29]) was utilized in the present study. Lastly, based on the relationship between regulatory focus and optimism/pessimism, corresponding associations with health outcomes are expected as well [30, 31]. Furthermore, regulatory focus orientation has been shown to be an important regulator of responses to health messages [32].

## Methods

### Participants and procedures

The study sample was acquired between December 2015 and February 2016 utilizing the online survey tool SoSciSurvey [33],after the design was met with approval by the ethics commission of the University of Applied Sciences Magdeburg-Stendal (AZ-3973-51). Participants were recruited for the study by means of social networks and bulletin boards. After receiving an introduction with regard to the general purpose of the study, participants gave their informed consent.

The total number of participants who started the survey by giving consent was *N* = 1173, of which *n* = 282 (24%) aborted the survey before answering all questions. Participants, who aborted the survey after providing their socio-demographic information but before completing any of the presented questionnaires (*n* = 149 [13%]) differed significantly from those who completed additional questionnaires in two of the six sociodemographic variables. Participants that aborted the survey were more likely to report a male gender (χ^2^(2) = 12.39, *p* = .002) and lower education (χ^2^(6) = 14.58, *p* = .024). Age (*U* = 68,923.00, *p* = .740), employment status (χ^2^(5) = 3.59, *p* = .610), family status (χ^2^(5) = 0.70, *p* = .983), and monthly net household income (χ^2^(8) = 6.23, *p* = .622) did not differ between participants who continued with the survey and those who did not. Due to the design of the online survey, either participants answered all items of a given scale or none at all; there was no missing data. Individuals who were too young to take part in the study (under the age of 18 years) were excluded. Thus, the used sample consisted of *n* = 1024. Participants who were included in the analysis had a mean age of around 30 years (*M* = 29.38; *SD* = 10.79) with a range from 18 to 70 years. Detailed sample characteristics are presented in Table 1.

**Table 1:** Sociodemographic characteristics of the study sample as well as means and standard deviations for the RFQ subscales, presented as M(SD)

	N	Percent	RFQ Promotion	RFQ Prevention
Gender				
Female	821	80.2	3.68 (0.58)	3.47 (0.79)
Male	196	19.1	3.67 (0.63)	3.24 (0.78)
Other	7	0.7	3.21 (0.50)	3.51 (0.74)
Age (years)				
≤ 20	160	15.6	3.62 (0.61)	3.49 (0.78)
21–30	543	53.0	3.67 (0.59)	3.46 (0.81)
31–40	163	15.9	3.69 (0.58)	3.34 (0.72)
> 40	158	15.4	3.73 (0.56)	3.29 (0.80)
Family status				
Single	572	55.9	3.63 (0.62)	3.44 (0.81)
Committed Relationship	236	23.0	3.72 (0.54)	3.47 (0.77)
Married	156	15.2	3.75 (0.55)	3.33 (0.73)
Separated	12	1.2	3.75 (0.42)	2.85 (0.71)
Divorced	40	3.9	3.75 (0.63)	3.26 (0.87)
Widowed	8	0.8	3.73 (0.55)	4.03 (0.47)
Education				
Pupil	32	3.1	3.52 (0.67)	3.59 (0.70)
≤ 8 years	28	2.7	3.33 (0.51)	3.14 (0.82)
9–11 years	130	12.7	3.47 (0.58)	3.21 (0.81)
≥ 12 years	834	81.4	3.73 (0.58)	3.46 (0.79)
Employment status				
Working full time	308	30.1	3.71 (0.58)	3.29 (0.77)
Working part time	141	13.8	3.62 (0.56)	3.42 (0.82)
Student/Apprentice	491	47.9	3.72 (0.59)	3.54 (0.79)
Unemployed	42	4.1	3.26 (0.60)	3.33 (0.86)
Homemaker	22	2.1	3.62 (0.58)	3.10 (0.59)
Retired	20	2.0	3.49 (0.63)	3.13 (0.69)
Monthly household net income				
< 1000 €	365	35.6	3.69 (0.60)	3.59 (0.77)
1000–1999 €	256	25.0	3.61 (0.58)	3.38 (0.82)
≥ 2000 €	325	31.7	3.71 (0.59)	3.29 (0.77)
No answer	78	7.7	3.68 (0.59)	3.43 (0.79)

### Measures

#### Regulatory focus questionnaire (RFQ)

The Regulatory Focus Questionnaire [17] comprises eleven items assessing the regulatory focus orientation of an individual. It includes six items for promotion focus and five items for prevention focus. Options for answering the items range from 1 – “never or seldom”/“never true”/“certainly false” to 5 – “very often”/“very often true”/“certainly true”. Seven of the items (1, 2, 4, 6, 8, 9, 11) need to be reverse-scored before calculating the respective mean scale scores. Higgins and colleagues [17] reported internal consistency as α = .73 for the promotion subscale and as α = .80 for the prevention subscale, and their intercorrelation was *r* = .21. It should be noted that values lower than α = .70 have been found before for the promotion subscale [34]. For the present study, two professional translators converted the original English version items into German independent of one another. After reaching a consensus on a single translation the items were translated back into English by two native speakers and compared with the original. Both language versions are displayed in Table 3. Scale characteristics for the German version are reported in the results section.

#### Behavioral inhibition/approach system scale (BIS/BAS)

The German BIS/BAS [35] was used to measure approach and avoidance motivation. It consists of 20 items, which are split among four subscales (*BIS*, *BAS-Drive, BAS-Fun Seeking*, *BAS-Reward Responsiveness*), and four filler items. Scale values are calculated by averaging item scores after reverse-scoring two of them. Strobel and colleagues [35] reported the internal consistency of the *BIS* scale as α = .78, and the *BAS* scale as α = .81.

#### Core self-evaluations scale (CSES)

The CSES ([36]) includes facets of self-esteem, locus of control, neuroticism, and self-efficacy in a 12 item scale. For the German version, evidence by Zenger and colleagues [25] suggested a two-factor interpretation. The, two scale scores are obtained by averaging the positively- and the negatively worded items, respectively. Internal consistency was reported as between α = .81 and .86.

#### Big five Inventory-10 (BFI-10)

The *Big Five* personality dimensions (*Openness*, *Conscientiousness*, *Extraversion*, *Agreeableness*, and *Neuroticism*) were measured using the BFI-10 [26]. Every subscale consists of two items, one of which has to be reverse-scored before calculating the mean. Rammstedt and John [26] reported test-retest-reliability as *r*_*tt*_ = .78 for *Openness*, *r*_*tt*_ = .83 for *Conscientiousness*, *r*_*tt*_ = .66 for *Agreeableness*, *r*_*tt*_ = .87 for *Extraversion*, and *r*_*tt*_ = .71 for *Neuroticism*.

#### Life orientation test – Revised (LOT-R)

The LOT-R [29] uses ten items to measure optimism and pessimism, four of which are filler items. A two-factor interpretation has been found to be preferable for the German version [37]. Scale scores for the two subscales are computed by adding individual item scores. Cronbach’s α was reported as α = .70 for the optimism scale and as α = .74 for the pessimism scale in the German general population [38].

#### Patient health Questionnaire-4 (PHQ-4)

The PHQ-4 [39] is an ultra-short screening instrument for symptoms of depression and anxiety using four items. Participants indicate to what extent they suffered from specific symptoms during the last two weeks. Summing up the items yields a scale score, measuring psychological distress. Löwe and colleagues [39] reported an internal consistency of α = .82 for the scale.

#### Somatic symptom Scale-8 (SSS-8)

The SSS-8 measures experienced somatic stress using eight items [40]. Adding all items provides a total score. The internal consistency of the scale was reported as α = .81 by Gierk and colleagues [40].

#### Subjective health status

From the EuroQol-5D [41], the visual analogue scale (VAS) was utilized to measure participants’ current subjective health status. It ranges from (0) “worst imaginable health status” to (100) “best imaginable health status”.

### Statistical analyses

Statistical operations were conducted using IBM SPSS Statistics 23 and AMOS 23. Pearson product-moment correlation coefficients were employed for reporting correlations. An α level of .05 was used for tests of significance unless otherwise noted. Properties of scales and items, such as means, standard deviations, item-difficulty indices as well as item-total correlations, were determined for the RFQ. Additionally, item and scale distributions were tested for normality by calculating skewness and kurtosis. The assumptions of sphericity and sampling adequacy were examined.

For the exploratory (EFA) and the confirmatory factor analyses (CFA), two randomly split subsamples of roughly the same size were used (n_EFA_ = 510; n_CFA_ = 514). The subsamples did not differ significantly in terms of age, gender, and RFQ item scores. For the EFA, principal component analysis (PCA), the minimum average partial (MAP; [42]) test, and parallel analysis (PA; [43]) were used. In the MAP test, the average squared partial correlations serve as indicators to determine the ideal number of factors. In PA, eigenvalues of randomly generated correlation matrices based on the original raw data (same number of variables and cases) are tested for significant differences from the empirically found ones. O’Connor [44] supplies SPSS syntaxes for these operations. Covariance matrices and the maximum likelihood method were used for the CFA.

The following model fit indices were used for the CFA with commonly agreed upon cut-off values [45–48]. The χ^2^-statistic and the minimum discrepancy divided by degrees of freedom (CMIN/DF) were used. Ideally, the former should be non-significant, although that rarely happens with larger sample sizes [49],while the CMIN/DF should be lower than five. The comparative fit index (CFI) and the Tucker-Lewis index (TLI) should be greater than .95, while a CFI/TLI that is greater than .90 can still be acceptable. The standardized root mean square residual (SRMR) should be lower than .08, although ideally lower than .06. Similar values are used for the root mean square error of approximation (RMSEA) and its 90% confidence interval. The Bayesian Information Criterion (BIC) is a comparative measure of fit and is utilized for comparisons between several models which do not necessarily have to be nested [50, 51]. Smaller BIC values indicate better fit. Raftery [51] reported guidelines for interpreting differences in BIC between models, suggesting that a margin of 10 between model BICs is the equivalent of a significant difference at the *p* = .001 level, given a sample size of at least 30. We use the BIC to compare between the two alternative models reported in the present study.

A multiple-group factor analysis was used to test for measurement invariance in a two-step process. Firstly, the unconstrained, configural model was compared with the metric model, which constrains unstandardized item loadings to be equal across groups. Secondly, the metric model and the scalar model, which constrains both, unstandardized item loadings and item intercepts, across groups, were compared. As per previous research, the differences in CFI and gamma hat (GH; [52]) were used as indicators for invariance along with the differences in the χ^2^ -statistic [53, 54]. A deviation of more than .01 in CFI or GH should be considered a sign of violations of measurement invariance.

Finally, differences in promotion and prevention focus across sociodemographic groups were tested for significance, for groups with at least 20 members. Normal distribution and equality of variances could be confirmed. In order to avoid an accumulation of α error probability, a significance level of .01 was utilized for the ANOVAs. Furthermore, Tukey’s HSD was used to compare individual groups for significant differences. Reported effect sizes are interpreted using Cohen’s *d* and η^2^, including 95% and 90% confidence intervals, respectively [55].

## Results

### Item characteristics and reliability

Item and scale characteristics are reported in Table 2. Skewness and kurtosis are well within the norms of having an absolute value of less than 1 for skewness and less than 3 for kurtosis [56]. Thus, a normal distribution can be assumed for all items and scales. Means and item-difficulty indices suggested that participants tended to answer items in the direction of the trait in question. Corrected item-total correlations ranged from .24 to .65. Usually, a value of .3 is considered a cut-off point for this coefficient [57]. The internal consistency of the subscales was ω = .78, 95%-CI = [.76; .80] for prevention focus, which is a good, and ω = .61, 95%-CI = [.58; .65] for promotion focus, which is mediocre and questionable [58]. After removing Item 3 the reliability of the promotion subscale did not worsen significantly, remaining at ω = .61, 95%-CI = [.57; .65]. This in conjunction with the poor item characteristics for Item 3 suggest its exclusion from the scale.

**Table 2:** Characteristics of the RFQ items and scales

Item/Scale	*M*(*SD*)	γ_1_	γ_2_	*P*	*r*_*it*_
RFQ 1^a^	3.81 (1.02)	−.54	−.36	.76	.40
RFQ 2^b^	3.02 (1.16)	.08	−.74	.60	.65
RFQ 3^a^	3.51 (1.00)	−.46	−.24	.70	.24
RFQ 4^b^	3.19 (1.18)	−.19	−.84	.64	.54
RFQ 5^b^	3.78 (0.88)	−.68	.26	.76	.51
RFQ 6^b^	3.60 (1.14)	−.47	−.61	.72	.55
RFQ 7^a^	3.65 (0.81)	−.56	.48	.73	.41
RFQ 8^b^	3.52 (1.08)	−.39	−.57	.70	.50
RFQ 9^a^	3.33 (1.11)	−.36	−.64	.67	.33
RFQ 10^a^	4.00 (0.89)	−.87	.85	.80	.38
RFQ 11^a^	3.75 (1.20)	−.70	−.50	.75	.34
RFQ Promotion^c^	3.71 (0.63)	−.41	.02		
RFQ Prevention	3.42 (0.79)	−.34	−.27		

^a^ = promotion item; ^b^ = prevention item; γ_1_ = skewness; γ_2_ = kurtosis; *P* = difficulty index; *r*_*it*_, = corrected item-total correlation. ^c^ Values reported for the promotion scale excluding Item 3

### Factor structure

The PCA of the first subsample (*n* = 510) using a Varimax rotation reduced the eleven items of the RFQ to two components: A prevention factor with an eigenvalue of 2.75 (explaining 25% of total variance) as well as a promotion factor with an eigenvalue of 2.18 (explaining an additional 20% of total variance). Similarly, the scree plot also indicated a distinct decline of explained variance after two factors. The intercorrelation of the extracted factors was *r* = .13. As reported in Table 3, factor loadings showed strong associations between all items and their respective factor. With the exception of Item 3, which loaded on its factor with .46, all items exhibited loadings of .60 and higher. The MAP test showed that the lowest average partial correlations between items could be found when assuming two factors. Likewise, the PA indicated that eigenvalues of factors one and two were larger than what could be expected with random data sets of the same number of variables and cases with a 95% margin of error. Thus, all methods of EFA suggested a two-factor solution (Table 4).

**Table 3:** Factor loadings of all RFQ items in the EFA

Item	German	English	Promotion	Prevention
RFQ 1	Sind Sie im Vergleich mit den meisten Menschen normalerweise nicht in der Lage, im Leben das zu erreichen, was Sie sich wünschen?	Compared to most people, are you typically unable to get what you want out of life?	.64	
RFQ 2	Haben Sie in Ihrer Kindheit jemals “Grenzen überschritten” indem Sie Dinge getan haben, die Ihre Eltern nicht duldeten?	Growing up, would you ever “cross the line” by doing things that your parents would not tolerate		.83
RFQ 3	Wie oft wurden Sie durch das Erreichen von Zielen dazu angespornt, noch härter zu arbeiten?	How often have you accomplished things that got you “psyched” to work even harder?	.46	
RFQ 4	Sind Sie Ihren Eltern während Ihrer Kindheit häufig auf die Nerven gegangen?	Did you get on your parents’ nerves often when you were growing up?		.71
RFQ 5	Wie häufig haben Sie Regeln und Vorschriften Ihrer Eltern befolgt?	How often did you obey rules and regulations that were established by your parents?		.68
RFQ 6	Haben Sie als Kind je ein Verhalten gezeigt, dass Ihre Eltern verwerflich fanden?	Growing up, did you ever act in ways that your parents thought were objectionable?		.73
RFQ 7	Haben Sie häufig Erfolg bei verschiedenen Sachen, die Sie ausprobieren?	Do you often do well at different things that you try?	.64	
RFQ 8	Ich bin schon manchmal in Schwierigkeiten geraten, weil ich nicht vorsichtig genug war.	Not being careful enough has gotten me into trouble at times.		.71
RFQ 9	Wenn ich Ziele erreichen will, die mir wichtig sind, sind meine Leistungen häufig nicht so gut wie ich es gerne möchte.	When it comes to achieving things that are important to me, I find that I don’t perform as well asI ideally would like to do.	.60	
RFQ 10	Ich habe den Eindruck, dass ich Fortschritte gemacht habe, was meinen persönlichen Erfolg im Leben angeht.	I feel like I have made progress toward being successful in my life.	.64	
RFQ 11	Ich habe sehr wenige Hobbys oder Interessen, für die ich mich begeistern kann oder die mich dazu motivieren, mich für sie anzustrengen.	I have found very few hobbies or activities in my life that capture my interest or motivate me to put effort into them.	.60	

Factor loadings smaller than .20 are not shown

**Table 4:** Results of the minimum average partial test and parallel analysis

	MAP test	PA Eigenvalues	
Factors	Average Squared Partial Correlations	Raw Data	Random data^a^
0	.052		
1	.033	2.748	1.307
2	.027	2.179	1.221
3	.045	.986	1.160
4	.064	.901	1.108
5	.103	.784	1.064
6	.155	.758	1.024
7	.212	.651	.987
8	.318	.604	.950
9	.467	.541	.909
10	1	.460	.868
11		.388	.823

^a^The random data represents the upper limit of the 95% confidence interval of the eigenvalue distribution of 1000 random data sets

The EFA clearly suggested a two-factor model. This model was subsequently tested in the CFA using the second subsample (*n* = 514). Model fit indices for models including and excluding Item 3 are reported in Table 5. The fit for the model including Item 3 was barely acceptable in terms of CFI and TLI, while showing good fit via SRMR and RMSEA. The exclusion of Item 3 led to sizable improvements across all fit indices. Furthermore, BIC clearly indicated that the model excluding Item 3 fit the data better than the original model. Loadings ranged between .54 and .74 for the prevention factor and between .41 and .52 for the promotion factor, except for Item 3, which loaded very weakly on its factor with .29. After removal of Item 3, the promotion factor loadings improved slightly to between .42 and .54. The correlation of the latent factors was *r* = .12 with and *r* = .15 without Item 3.

**Table 5:** Model fit indices of the calculated two factor models

Model	χ^2^(*df*)	*p*	CMIN/DF	CFI	TLI	RMSEA [90% CI]	SRMR	BIC
Including RFQ 3	103.08 (43)	< .001	2.397	.928	.908	.052 [.039; .065]	.053	246.65
Excluding RFQ 3	73.14 (34)	< .001	2.151	.951	.934	.047 [.032; .062]	.046	204.23

*CMIN/DF* minimum discrepancy divided by degrees of freedom, *CFI* comparative fit index, *TLI* Tucker-Lewis index, *RMSEA* root mean square error of approximation including 90% confidence interval, *SRMR* standardized root mean square residual, *BIC* Bayesian Information Criterion

The analysis of measurement invariance revealed clear evidence for metric invariance across males and females as well as across age groups, as neither the χ^2^-statistic nor the CFI or the GH indicated statistically significant differences (or just barely significant differences in the case of the χ^2^-test for age groups). Scalar invariance however could not be confirmed unequivocally. The differences in the GH index for both comparisons were not larger than .01, however both the CFI and the χ^2^-test indicated significant differences between models (Table 6).

**Table 6:** Fit indices for the multigroup analysis

Model	χ^2^(*df*)	Δ χ^2^	Δ *p*	CFI	ΔCFI	GH	ΔGH
Gender							
Female	123.92 (34)			.937		.978	
Male	71.99 (34)			.888		.962	
Multigroup analysis							
Configural invariance	196.04 (68)			.928		.975	
Metric invariance	202.00 (76)	5.96	.652	.929	.001	.976	.001
Scalar invariance	237.96 (86)	35.96	.001	.914	.015	.971	.005
Age, years							
≤ 20	55.09 (34)			.926		.975	
21–30	80.74 (34)			.957		.983	
31–40	63.09 (34)			.890		.965	
> 40	100.37 (34)			.736		.921	
Multigroup analysis							
Configural invariance	436.35 (198)			.874		.956	
Metric invariance	446.52 (206)	10.17	.254	.873	.001	.954	.002
Scalar invariance	477.40 (216)	30.88	.001	.862	.011	.952	.002

*CFI* comparative fit index, *GH* gamma hat

### Validity

The RFQ Promotion and the RFQ Prevention scales were correlated with the conceptually related scales mentioned in the Introduction in order to examine the construct validity of the RFQ. These scales include: a behavioral-motivational scale (BIS/BAS), a core self-evaluation questionnaire (CSES), a personality scale (BFI-10), an instrument measuring optimism and pessimism (LOT), as well as three short questionnaires assessing somatic and mental health-related constructs (PHQ-4, SSS-8, Health VAS). Correlation coefficients are presented in Table 7.

**Table 7:** Correlations between the RFQ scales and further psychological measures

	RFQ Promotion Focus	RFQ Prevention Focus
BIS (Inhibition)	−.34 **	.12 **
BAS (Activation)	.34 **	−.14 **
CSES positive	.66 **	.07 *
CSES negative	−.48 **	−.07 *
BFI-10 Openness	.20 **	.00
BFI-10 Conscientiousness	.32 **	.17 **
BFI-10 Extraversion	.33 **	−.11 **
BFI-10 Agreeableness	.14 **	.12 **
BFI-10 Neuroticism	−.36 **	.04
LOT-Optimism	.50 **	.05
LOT-Pessimism	−.60 **	−.15 **
PHQ-4	−.46 **	−.09 **
SSS-8	−.33 **	−.16 **
Subjective health status (VAS)	.33 **	.08 *

*p < .05; **p < .01. The Promotion scale excludes Item 3

### Differences based on socio-demographic variables

Means and standard deviations of all compared groups are presented in Table 1. Women were found to be significantly more prevention-oriented than men, *t*(1015) = 3.63, *p* < .001, *d* = 0.29, 95% *CI* [0.13; 0.46]. However there was no difference with regard to promotion focus, *t*(1015) = −.104, *p* = .917, *d* = 0.02, 95% *CI* [−0.18; 0.15].

Age groups did not differ significantly in terms of promotion focus, *F*(3,1020) = 3.17, *p* = .024, η^2^ = .01, 90% *CI* [< 0.01; 0.02], and prevention focus *F*(3,1020) = 2.94, *p* = .032, η^2^ = .01, 90% *CI* [< 0.01; 0.02]. None of the post-hoc comparisons were significant.

There were no significant differences across groups of family status for either promotion, *F*(3,1000) = 3.52, *p* = .015, η^2^ = .01, 90% *CI* [< 0.01; 0.02], or prevention focus, *F*(3,1000) = 1.76, *p* = .154, η^2^ = .01, 90% *CI* [< 0.01; 0.01]. None of the post-hoc comparisons were significant.

Groups of various levels of net household income differed significantly with regard to their prevention focus, *F*(3,1020) = 8.90, *p* < .001, η^2^ = .03, 90% *CI* [0.01; 0.04], but not in terms of their promotion focus, *F*(3,1020) = 2.42, *p* = .065, η^2^ = .01, 90% *CI* [< 0.01; 0.02]. Post-hoc tests revealed that the low income group scored higher on the prevention scale than the moderate income groups, *p* = .008, *d* = 0.27, 95% *CI* [0.11; 0.43], and also higher than the high income groups, *p* < .001, *d* = 0.39, 95% *CI* [−0.24; 0.54].

Finally, there were significant differences in both promotion focus, *F*(5,1018) = 4.24, *p* = .001, η^2^ = .02, 90% *CI* [0.01; 0.03], and prevention focus, *F*(5,1018) = 5.49, *p* < .001, η^2^ = .03, 90% *CI* [0.01; 0.04], across groups of employment status. Post-hoc tests showed the differences in promotion focus to be between unemployed participants and those working full time, *p* < .001, *d* = 0.77, 95% *CI* [0.45; 1.10] between unemployed participants and those working part time, *p* = .004, *d* = 0.63, 95% *CI* [0.28; 0.98], as well as between unemployed participants and those in training/education, *p* < .001, *d* = 0.78, 95% *CI* [0.46; 1.10]. Unemployed participants showed the lower scores in all of these comparisons. Differences in prevention focus were found between those working full time and those in training/education, *p* < .001, *d* = 0.32, 95% *CI* [0.18; 0.46], with those in full time employment displaying lower prevention focus.

## Discussion

The aim of the present study was to translate the RFQ into German, to test its psychometric properties, and examine aspects of validation. All items showed good psychometric properties with the exception of Item 3, which displayed a poor correlation with the total scale score. Additionally, factor loadings in EFA as well as in CFA were good for all items except Item 3. Higgins and colleagues [17] had found a similarly small factor loading of .37 for Item 3 on the promotion factor. Therefore, despite the original intention of making the German version of the scale as comparable as possible to the English version, Item 3 had to be excluded from the scale. Reliability coefficients were good for the prevention scale and questionable for the promotion scale, even with the exclusion of Item 3. Previous research suggests that the application of translated questionnaires in different countries or cultures can lead to a decline in reliability, especially when reverse-scored items are used [59, 60]. This could explain the present findings with regard to the promotion scale and Item 3. To put these findings in perspective, it is important to note that even with a reliability as low as .60, strong correlations of up to *r* = .77 towards other constructs are possible, as evidenced by the high correlations of the promotion scale with the CSES and the LOT.

Fit indices for the two-factor model including Item 3 indicated acceptable fit. However, there was a decided improvement of the fit between data and model, when Item 3 was removed from the promotion scale. Weak factorial (or metric) measurement invariance could be shown for gender as well as age groups. Although strong factorial (or scalar) measurement invariance was indicated for both groups by the acceptable deviation in GH, this evidence is ambiguous because of the larger than .01 difference in CFI between models. Measurement invariance would suggest that participants across groups respond to the given items in a comparable manner with regard to the latent construct. Thus, it is important to unambiguously confirm or reject scalar invariance of the measure in a more representative sample of the general population. We suspect this potential deviation from invariance to be founded in the wording of the original English items, for which there was never an analysis of measurement invariance.

Largely, we could find the expected pattern of correlations for the promotion subscale, but only in part for the prevention subscale. Overall, the promotion scale had moderate to strong associations with most of the employed questionnaires, suggesting good convergent validity. Correlations for the prevention scale were however much lower than they were for the promotion scale. This is in line with previous research consistently showing higher correlations for promotion than for prevention focus [61, 62]. We suspect that these low associations might be due to the prevention focus scale’s focus on a person’s childhood experiences as opposed to current personality traits. This is also a crucial limitation to not just the German version of the scale but the RFQ in general. As predicted, promotion focus correlated positively with behavioral activation and negatively with behavioral inhibition, while prevention focus correlated negatively with behavioral activation and positively with behavioral inhibition. This replicates the findings of Summerville and Roese [15], who found very similar correlations. Promotion focus was shown to be a very good predictor for evaluations of self-esteem and capabilities, as evidenced by the correlation with the CSES. Furthermore, promotion focus displayed relations with all dimensions of the BFI – not just openness and extraversion -, while prevention focus was only moderately associated with Conscientiousness, Extraversion, and Agreeableness. In keeping with Hazlett and colleagues [34], promotion focus correlated negatively with pessimism and positively with optimism, while prevention focus did not show the expected associations. Finally, promotion as well as prevention focus were associated with health-related outcomes, although the (weak negative) correlation of prevention focus was not expected.

Several differences in regulatory focus based on sociodemographic variables became apparent. Firstly, females were found to be significantly more prevention focused then men. Secondly, individuals with a lower monthly net household income exhibited higher prevention focus than those with higher incomes. Finally, unemployment was related to lower promotion focus, while students/apprentices showed higher prevention focus than those working full time. The differences in regulatory focus across employment status groups correspond to a moderately large effect. This is a very interesting finding and may warrant further investigation. Regulatory focus could therefore play a role in developments leading to unemployment or unemployment could lead to a decline of promotion focus.

Regulatory focus is an important construct in personality and social psychology and is highly relevant towards important domains such as work-related outcomes. Therefore, the RFQ can be recommended for application in all fields dealing with motivation and goal attainment processes.

### Limitations

When comparing the sample with population averages obtained from the Federal Statistical Office of Germany [63], it became clear that representativeness can not be assumed. The present study sample was relatively young. In addition, women are over- and men under-represented. Furthermore, the sample was more educated than expected in the general population, with more than 80 % reporting a university entrance qualification, compared to roughly 30 % in the general population. Household net income was reported as lower than the population average, which could also be because of the high number of singles and young people in the sample. Lastly, study participants were more likely to be students or apprentices, and less likely to be working, unemployed, staying at home, or retired. Therefore, the RFQ should be examined with a more representative sample in further studies in order to establish norm values.

Strong measurement invariance could not be shown unambiguously. There is clear evidence for metric invariance for gender and age groups but full scalar invariance could not be demonstrated beyond a doubt. Therefore, the comparisons between sociodemographic groups should be interpreted with caution. Further analysis in representative samples is recommended.

In terms of convergent validity, it became clear that especially the prevention focus subscale warrants further investigation, as it showed weak to moderate correlations across the board, despite good psychometric properties, such as high reliability – especially when taken in context of the high correlations the promotion subscale achieved in spite of its low reliability.

Finally, the present study is entirely based on cross-sectional self-reports. Therefore, we can not make any predictions with regard to behavior apart from the association with other personality measures.

## Conclusion

Overall, the RFQ is a measure of regulatory focus that shows acceptable reliability and good validity towards related psychological constructs. Factor structure and fit between data and theoretical model were very good. Therefore, the German RFQ can recommended for use in research of regulatory focus and practical applications.
